# HIV associated hypocalcaemia among diarrheic patients in northwest Ethiopia: a cross sectional study

**DOI:** 10.1186/1471-2458-14-679

**Published:** 2014-07-04

**Authors:** Beyene Moges, Bemnet Amare, Timoki Yabutani, Afework Kassu

**Affiliations:** 1 Department of Immunology and Molecular Biology, School of Biomedical and Laboratory Sciences, College of Medicine and Health Sciences, University of Gondar, Gondar, Ethiopia; 2 Department of Biochemistry, School of Medicine, College of Medicine and Health Sciences, University of Gondar, Gondar, Ethiopia; 3 Department of Chemical Engineering, University of Tokushima, Tokushima, Japan; 4 Department of Medical Microbiology, School of Biomedical and Laboratory Sciences, College of Medicine and Health Sciences, University of Gondar, Gondar, Ethiopia

**Keywords:** Hypocalcaemi, Hypercalcaemia, HIV, Diarrhea, Ethiopia

## Abstract

**Background:**

Hypocalcaemia, defined by serum calcium level less than 8.5 mg/dl, could be caused by human immunodeficiency virus (HIV) and diarrheal diseases. In Ethiopia, while morbidities from diarrheal diseases and HIV are serious health problems, studies assessing the interactions amongst of the three do not exist. Therefore, the present study was undertaken to investigate the level of calcium among diarrheic patients with and without HIV co-infection.

**Methods:**

**C**onsecutive diarrheic patients attending Gondar University Hospital in Ethiopia were enrolled and screened for HIV, intestinal parasites, *Shigella* and *Salmonella*. Concentration of calcium in serum was determined using an inductively coupled plasma mass spectrometer.

**Results:**

A total of 206 diarrheic patients were included in the study (109 = HIV positive, 97 = HIV negative). Intestinal parasites and *Shigella* species were detected in 32.2% and 8.5% of the patients, respectively. The serum calcium levels in the patients who were found positive for *Shigella* species or intestinal parasites was not significantly different by the presence or absence of HIV co-infection. HIV infected diarrheic patients had significantly lower mean serum calcium levels (7.82 ± 1.23 mg/dl) than those negative for HIV (8.38 ± 1.97) (P = 0.015). The age groups 25–35 and greater than 45 years showed significantly lower mean serum calcium levels (7.77 ± 1.55 mg/dl) in comparison to the other age groups (7.84 ± 1.41 mg/dl, P = 0.009). On the other hand, females presented with significantly lower mean serum calcium levels (7.79 ± 1.60 mg/dl, P = 0.044) than males (8.26 ± 1.65 mg/dl).

**Conclusion:**

There is high prevalence of hypocalcaemia among diarrheic patients in northwest Ethiopia. And HIV stood out to be a major risk factor for development of hypocalcaemia among the diarrheic patients in northwest Ethiopia. Further studies are required to substantiate and characterize the mechanisms and consequences of calcium metabolism disorders among HIV infected individuals in the study area.

## Background

Diarrheal diseases, intestinal parasitic infections and enteropathogenic bacteria represent one of the six leading causes of death worldwide [1] with an enormous diseases burden associated with them [2,3]. They are also one of among the important causes of morbidity and mortality in developing countries [4]. The situation is severe in sub-Saharan Africa, a region where an estimated 25.8 million adults and children are infected with human immunodeficiency virus (HIV) [5]. HIV manifests with chronic [4] and persistent diarrhea in which more than half of the cases is due to intestinal parasites [6-9].

Disturbances of electrolyte metabolism and endocrine regulation like hyponatraemia [10-12], hypo- and hyperkalaemia [13-18] have been observed in HIV patients. In addition, lactic acidosis [19], hypophosphatemia, [20], hypernatremia [21], and hypocalceamia [20,22] have also been described. In a recent study conducted by our group in northwest Ethiopia, hypercalcemia was widespread among asymptomatic HIV infected patients [23].

Calcium is required for vascular contraction and vasodilation, muscle function, nerve transmission, intracellular signaling and hormonal secretion. However, less than 1% of total body calcium is needed to support these critical metabolic functions [24]. Hypocalcaemia, defined by serum calcium level less than 8.5 mg/dl [25], could be caused by HIV infection [20,22,26]. Diarrheal diseases could also cause hypocalcaemia through malabsorption and sepsis [27-30]. In Ethiopia morbidities from diarrheal diseases and HIV are serious health problems. However, studies assessing the interactions between diarrheal diseases, HIV and calcium metabolism do not exist. Therefore, the present study was undertaken to investigate the level of calcium among diarrheic patients with and without HIV co-infection.

## Methods

Consecutive diarrheic patients attending Gondar University Hospital in Ethiopia were enrolled. The University teaching hospital is a major tertiary levels referral hospital rendering health services for over 5 million inhabitants in the Northwest Ethiopia. All HIV-infected patients were naive to antiretroviral drugs at the time of data collection. Patients with hyperparathyroidism and other known calcium metabolism disorders were excluded from the analysis.

### Stool examination and culture

Stool specimens were collected following the standard procedure [30]. Samples were then inoculated immediately on MacConkey and Salmonella-Shigella agar plates (Oxoid). The inoculated plates were incubated at 37°C aerobically for 24 hours. The plates were then examined for the presence or absence of visible bacterial colonies. The presence of non-lactose fermenting (NLF) colonies was taken as a presumptive diagnostic tool for *Shigella* and *Salmonella* species. The NLF colonies were further tested through a series of biochemical tests followed to identify *Shigella* and *Salmonella* species [31]. Proper microbiological quality control was employed at each step of the procedure and American Type Culture Collection quality control strains of Escherichia coli (ATCC 25922) and *Pseudomonas aeruginosa* (ATCC 27853) were used. Stool specimens were also processed and examined by direct microscopy for intestinal parasites. Modified acidfast staining technique was also employed to detect *Cryptosporidium parvum* and *Isospora belli*[32].

### Blood collection, clinical chemistry and HIV serology

Blood specimens were taken with minimal venostasis after overnight fasting for the measurement of serum calcium from diarrheic patients. The presence of HIV antibodies was determined by an enzyme linked immunosorbent assay following the manufacturer’s instruction (Vironostica HIV Uni-Form II plus O, Organon Teknika, Boxtel, the Netherlands). The concentration of albumin was determined photometrically (AUTOLAB PM 4000/3, Analyser Medical System, Italy). Hypoalbuminemia was defined as serum albumin level below 3.5 gm/dl [33].

### Determination of calcium in serum

The frozen serum samples were kept on dry ice and air freighted to Japan. Concentration of calcium in serum was determined using an inductively coupled plasma mass spectrometer (ICP-MS) (model 8500, Schimadzu, Tokyo, Japan), at Department of Analytical Chemistry, the University of Tokushima, Japan [34]. In brief, serum sample (200 μl) was aliquoted in to teflon tube and covered with teflon ball. After adding 1 ml of concentrated HNO_3_ (Wako Pure Chemicals, Japan), the tube was heated on an aluminum heating block (IWAKI, Asahi Techno Glass, Japan) at 120°C for 5 h. The sample was further heated almost to dryness at 200°C after removing the teflon ball. Finally, the residue was dissolved with 2 ml of 0.1 M HNO_3_ which contained 10 ng/ml internal standard elements (In, Re and Tl). The diluted serum solution was used for analysis of the calcium in ICP-MS. Commercially available single element standard solutions (1000 mg/ml) were purchased from Wako Pure Chemicals (Osaka, Japan) and used for standardization of calibration curves. To allow for protein binding of calcium, measured serum total calcium concentrations were corrected for hypoalbuminemia using the following equation as published by [35]: Corrected Ca = serum Ca + 0.8 (4 - serum albumin).

### Statistical analysis

Data were analyzed using SPSS version 16 statistical package. A one-sample Kolmogorov– Smirnov test was used to assess whether the data were normally distributed. Serum calcium values were log transformed for analysis. Comparisons of serum values of calcium among diarrheic patients with and without HIV co-infection versus shigellosis/intestinal parasitoses groups were made using a one-way ANOVA. Post-hoc Tukey test was used to determine which pairs of means differ significantly. The independent *T*-test was used to compare means among different groups of diarrheic patients. Logistic regression and multinomial regression models were used to check for statistical association between dependent and independent variables. Those variables which were found significantly associated with the dependent variables were further tested by controlling with other independent variables if association is still maintained. Hypoclacemia was defined as its serum levels less than 8.5 mg/dl while hypercalcemia was defined at its serum levels greater than 10.5 mg/dl [25]. *P-values* less than 0.05 were considered statistically significant.

### Ethical considerations

The study was conducted after ethical approval was obtained from Institutional Review Board of the University of Gondar and the University of Tokushima, Tokushima, Japan and after informed consent was obtained from adult study participants or legal guardians of children. Positive patients for shigellosis and intestinal parasitoses were treated following the nation’s standard clinical management protocols.

## Result

A total of 206 diarrheic patients were included in the study; out of which 109 (52.9%) were with HIV infection, 47 (22.8%) were with isolated shigellosis/intestinal parasitoses, and 50 (24.3%) were without HIV, shigellosis, and intestinal parasitoses infection. Among the 109 HIV positive participants 76 (69.7%) were having only HIV infection while 33 (30.3%) were having HIV-shigellosis/intestinal parasitoses co-infection.

Table 1 shows the demographic and clinical characteristics of the patients versus their mean serum calcium levels. The mean ± SD serum calcium (mg/dl) of the participants was 8.08 ± 1.60. Majority 128 (62.1%) of the participants had hypocalcaemia while 14 (6.8%) patients had developed hypercalcemia. The mean serum calcium levels varied significantly among different age groups, sex and HIV serostatus (P < 0.05) while other variables didn’t show any difference (Table 1). The age groups 25–35 and greater or equal to 45 years showed significantly lower mean serum calcium levels in comparison to the other age groups with 7.77 ± 1.55 mg/dl and 7.84 ± 1.41 mg/dl, respectively (P = 0.009). Among those with hypocalcaemia, 83.3% and 81.8% of those between 25–35 and > 45 years, respectively, had HIV infection. On the other hand females presented with significantly lower mean serum calcium levels (7.79 ± 1.60 mg/dl) than males (8.26 ± 1.65 mg/dl) (P = 0.044). Among those with hypocalcaemia, 79.5% and 70.8% of women and men had HIV infection, respectively. Having HIV was also associated with significantly lower mean serum calcium levels in comparison with not having HIV infection with 7.82 ± 1.23 mg/dl (P = 0.015) (Table 1).

**Table 1:** Demographic and clinical data and serum calcium levels of diarrheic patients in Gondar, Ethiopia

**Parameters**	**Serum calcium level**					
	**Normocalcemia N = 64 (31.1%)**	**Hypocalcaemia N = 128 (62.1%)**	**Hypercalcemia N = 14 (6.8%)**	**Total N = 206**	**Mean ± SD calcium**	P value*
**Age (years)**						0.009
**15-24**	23(35.9)	22(17.2)	7(25.2)	52(25.2)	8.72 ± 1.83	
**25-34**	20(31.2)	61(47.7)	3(21.4)	84(40.8)	7.77 ± 1.55	
**35-44**	15(23.4)	28(21.9)	3(21.4)	46(22.3)	8.05 ± 1.52	
**> = 45**	6(9.4)	17(13.3)	1(7.1)	24(11.7)	7.84 ± 1.41	
**Sex**						0.044
**Male**	42(65.6)	73(57)	12(85.7)	127(61.7)	8.26 ± 1.65	
**Female**	22(34.4)	55(43)	2(14.3)	79(38.3)	7.79 ± 1.60	
**Shigellosis**						0.857
**Yes**	4(6.)	11(8.6)	3(21.4)	18(8.7)	8.15 ± 2.15	
**No**	60(93.8)	117(91.4)	11(78.6)	188(91.3)	8.08 ± 1.59	
**Intestinal parasitoses**						0.672
**Yes**	22(34.4)	40(31.2)	4(28.6)	66(32)	8.01 ± 1.90	
**No**	42(65.6)	88(68.8)	10(71.4)	140(68)	8.12 ± 1.51	
**HIV serostatus**						0.015
**Yes**	24(37.5)	81(63.3)	4(28.6)	109(52.9)	7.82 ± 1.23	
**No**	40(62.5)	47(36.7)	10(71.4)	97(47.1)	8.38 ± 1.97	

*P value is calculated from independent *T*-test and one way ANOVA.

N = number, HIV = human immunodeficiency virus, SD = standard deviation.

Table 2 describes the albumin adjusted serum calcium levels (mg/dl) among diarrheic patients with and without HIV versus shigellosis and intestinal parasitoses. The mean ± SD serum calcium level of those only with HIV infection and with HIV-shigellosis/intestinal parasitoses co-infection were 7.76 ± 1.29 mg/dl and 7.96 ± 1.09 mg/dl, respectively. These low mean serum calcium levels were significantly lower than those without HIV infection (P = 0.021) (Table 2).

**Table 2:** Serum levels of calcium (mg/dl) in diarrheic patients with HIV and without HIV versus shigellosis and intestinal parasitoses in Gondar, Ethiopia

		**HIV (N = 76)**	**HIV/shigellosis/IP (N = 33)**	**Shigellosis/IP only (N = 47)**	**Negative for all**^**b**^**(N = 50)**
Mean ± SD*		7.76 ± 1.29	7.96 ± 1.09	8.06 ± 2.40	8.67 ± 1.42
**Median (Range)**		7.62(4.62-11.99)	8.03(6.26-10.68)	8.3(0.19-12.28)	8.58(3.16-12.08)
**Cut-off value no (%)**	**<8.5mg/dl**^**a**^	57(75)	24(72.7)	25(53.2)	22(44)
	**8.5-10.5mg/dl**	16(21.1)	8(24.2)	16(34)	24(48)
	**>10.5mg/dl**	3(4)	1(3.1)	6(12.8)	4(8)

^a^Cutoffs according to [25].

^b^Negative for HIV, Shigellosis and intestinal parasitoses.

IP = Intestinal Parasitoses, HIV = human immunodeficiency virus, SD = standard deviation, N = number.

*P = 0.021 using one way ANOVA.

Hypocalcaemia was found in 57 (75%) of those having HIV, in 24 (72.7%) of those with HIV-shigellosis/intestinal parasitoses co-infection, and in 25 (53.2%) of those with shigellosis/intestinal parasitoses infections. Among the diarrheic patients without HIV and shigellosis/intestinal parasitoses, 22(44%) developed hypocalcaemia (Table 2). Regardless of co-infection with shigellosis or intestinal parasitoses, hypocalcaemia was found among 81 (74.3%) of those with HIV infection. Using the multinomial regression model, HIV infection was found to be significantly associated with hypocalcaemia (OR = 4.31, 95% CI = 1.28-14.51). This statistical association persisted even after controlling for other characteristics like sex, age and shigellosis/intestinal parasitoses when separately added in the model (OR = 4.27, 95% CI = 1.25-14.53; OR = 3.68, 95% CI = 1.05-12.86; and OR = 3.89, 95% CI = 1.12-13.51, respectively). Similarly, the association persisted when only two other variables were combined and added in the model (P < 0.05). However, the association was lost when all parameters were added together to the model (P > 0.05).

Among the 14 diarrheic patients with hypercalcemia, 3 (21.4%), 1 (7.1%), 6 (42.9%), and 4 (28.6%) were with isolated HIV; HIV-shigellosis/intestinal parasitoses co-infection; isolated shigellosis/intestinal parasitoses; and without HIV, shigellosis or intestinal parasitoses, respectively (Table 2). The development of hypercalcaemia didn’t show any statistical association with any of the independent variables.

## Discussion

Apart from the common hyponatremia, hypo- and hyperkalemia [10-21], hypocalcaemia is recently reported as one of the multiple HIV associated electrolyte metabolism disturbances [20,22]. Generally hypocalcaemia has not been considered to be a frequent phenomenon in HIV infection and used to be mostly attributed to hypoalbuminaemia or to pharmacotherapy like foscarnet or ketoconazole [35-38]. In spite of this, the current study revealed high prevalence of hypocalcaemia in HIV infected participants (74.3%) than their negative counterparts (44%). The prevalence of the hypocalcaemia in the current study is far much higher than that reported from Germany (6.5%) [23]. One possible explanation for the high prevalence of hypocalcaemia among Ethiopia patients could be a dietary calcium deficiency (ascribed to low consumption of dairy products and a high-fibre diet). Another possible explanation might be the dark skin pigmentation of the African people leading to decreased dermal synthesis of vitamin D. Hypocalcaemia among HIV infected individuals could lead to osteoporosis and other related bone disorders. This, however, demands further longitudinal investigations.

As reflected by reports from several studies, the causes of hypocalcaemia in HIV infected patients could be multifactorial and likely represent a complex interaction between HIV infection, traditional hypocalcemic risk factors exacerbated by consequences of chronic HIV infection (eg, poor nutrition), low vitamin D levels and drug related factors [37,39-46]. In the current study, however, all the HIV infected participants were naïve to antiretroviral therapy and didn’t receive foscarnet or ketokonzole ruling out the effect of treatment associated hypocalcaemia. We also excluded all patients with known calcium metabolism disorders before the commencement of the study. On the other hand, renal failure, pancreatitis, malabsorption and sepsis have also been described as possible causes of hypocalcaemia during HIV infection [28-30].

Direct effects of HIV on the differentiation or activation of osteoclasts could be one more possible explanation for the hypocalcaemia. It is reported that HIV-1, Vpr, enhances production of receptor of activated NF-kappa B ligand (RANKL) via potentiation of glucocorticoid receptor activity [47] which activates formation of osteoclasts and inhibits osteoclast apoptosis [48]. On the other hand, persistent HIV infection or episodes of opportunistic infections have been shown to result in chronic T-cell activation and a pro-inflammatory cytokine milieu [49,50] that induces functionally active osteoclasts by expressing both a cell-bound and a soluble form of RANKL [51,52]. Of note, RANKL gene expression is enhanced by cytokines such as interleukin-1 (IL-1) and tumor necrosis factor alpha (TNF-α), which are elevated in HIV infection [48,51]. Moreover, IL-1 and TNF-α are capable of directly inducing differentiation and activation of osteoclasts in the absence of RANKL [53,54]. In the current study, HIV infection was significantly associated (OR = 4.31, 95% CI = 1.28-14.51) with hypocalcaemia; direct effects of the virus could be the primary mechanism for the hypocalcaemia. However, further studies are required to substantiate the causes and associated factors with hypocalcaemia in HIV patients in the study area.

On the other hand, in the current study, 8.7% of the participants had shigellosis that could result in to sepsis. It has been suggested that hypocalcaemia could be the result of an increased in IL-1 and TNF-α production, although the underlying mechanisms are unclear [54,55]. Despite its potent ability to stimulate bone resorption, IL-1 induces hypocalcaemia in mice and in rats [54,56], leading to the hypocalcaemia seen during sepsis [54,56]. Lipopolysaccharide (LPS), a component of the cell-walls of Gram negative bacteria like *Shigella* species, may stimulate many biological activities in a wide variety of cells via IL-1 and TNF [57]. Injection of sub-lethal doses of LPS into wild type mice by *Deng et al.*[58] also induced hypocalcaemia, increased IL-1, and increased TNF-α. A time-lag between IL-1- and TNF-α-stimulated Ca^2+^-entry into cells throughout the body from the circulation and IL-1-stimulated Ca^2+^-release from the bone was suggested to cause the observed LPS-induced hypocalcaemia [59]. However, isolated shigellosis was not found to be a risk factor for hypocalcaemia in the current study.

Bone is composed of matrix and osteoid, and it is mineralized with calcium and phosphate in the form of calcium hydroxyapatite. When bone mineralization is decreased, osteopenia can occur. Eventually, osteoporosis (i.e., porous bone) can result. This is seen pathologically as the structural deterioration of bone and can lead to nontraumatic fractures. Osteomalacia (i.e., soft bone) is most commonly the result of vitamin D deficiency and occurs when intact bone matrix is not adequately mineralized [60]. Among the validated risk factors recognized for fragility fracture in population studies is an increasing age [61,62]. In the current study, however, apart from the elderly with age greater than 45 years (mean serum calcium = 7.84 ± 1.41 mg/dl), the age groups 25–35 years showed significantly lower mean serum calcium levels (7.77 ± 1.55 mg/dl) in comparison to the other age groups (P = 0.009) (Table 1). This low serum calcium levels in the elderly could be due to aging itself which could further be exacerbated by HIV infection as 81.8% of them have HIV infection. In the age group 25–35 years, however, the low mean serum calcium levels could mainly be due to HIV as 83.3% of them had HIV infection. Low bone mineral density (BMD) has been reported in many cross-sectional studies involving younger [62] and older [63,64] HIV-infected individuals. Peak bone mass is achieved during adolescence and young adulthood and is a key determinant of bone mass in later life [65]. Thus, the effect of HIV infection and/or antiretroviral treatment (ART) on this process is a critical area of research.

In the HIV-negative people, BMD increases until around age 30 where it remains stable for perhaps 5–10 years before starting to decline (at a rate of 0.5-1% per year), especially, in women during menopause (during which it declines at −2% bone volume a year) [66]. Women generally have lower BMD than men [67]. In line with this, in the current study, females presented with significantly lower mean serum calcium levels than males with 7.79 ± 1.60 mg/dl (P = 0.044). Among those with hypocalcaemia, 79.5% and 70.8% of women and men had HIV infection, respectively.

Our study also revealed that 6.8% of the diarrheic patients had hypercalcaemia. Four (28.6%) of them were with HIV infection. This is in contrast to a report by our group which showed 56.3% of hypercalcemia among asymptomatic HIV infected individuals in northwest Ethiopia [25]. However, the participants in the current study were diarrheic patients which might play its part to the reduced serum calcium level. The mechanism of HIV associated hypercalcemia is not well established but Acquired immunodeficiency syndrome (AIDS)-related opportunistic infections (OIs) may lead to hypercalcemia. Infection with *Pneumocystis carinii*[68], *Mycobacterium avium*[69], lymphoma [70], *Cryptococcus neoformans* and *Coccidioides immitis*[71,72], Candidiasis and paracoccidioidomycosis [73] and concurrent Epstein-Barr virus infection [74] have all been reported to be associated with hypercalcemia in HIV patients though we didn’t detect any of them in the current study. It is interesting to note that ART–induced immune reconstitution may lead to the possibility of hypercalcemia [75,76]. The roles of cytokines, OIs, ART-associated immune reconstitution inflammatory syndrome in the pathogenesis of hypercalcemia among HIV/AIDS patients require further investigation.

In summary, there was a high prevalence of hypocalcaemia among diarrheic patients in northwest Ethiopia. HIV was a significant factor for development of hypocalcaemia in the study area. HIV associated malabsorption, sepsis, and direct effect of the virus on calcium metabolism could be the mechanisms of hypocalcaemia. However, further studies are required to substantiate how HIV is associated with a high prevalence of hypocalcaemia in the study area.

### Limitation of the study

The fact that the study was a cross sectional study it restricted the generalization we made. We didn’t also measure the serum Vitamin D level as its deficiency is a well established risk factor for hypocalcaemia though there is a reportedly high rate of biochemical vitamin D deficiency among Ethiopians [77] which could partly be associated with the dark skin pigmentation leading to decreased dermal synthesis of vitamin D [78]. We didn’t also measure the BMD of the participants to substantiate the long term effect of hypocalcaemia among HIV patients in the study area. Therefore, we suggest conducting further studies by addressing all the aforementioned gaps.

## Conclusion

There is high prevalence of hypocalcaemia among diarrheic patients in northwest Ethiopia. HIV stood out to be a major risk factor for development of hypocalcaemia among the diarrheic patients. Direct effects of the virus on calcium metabolism, sepsis, and malabsorption could be the mechanisms of hypocalcaemia. The high prevalence of the hypocalcaemia among HIV infected individuals could result in to bone disorders. However, further studies are required to substantiate and characterize the mechanisms and consequences of calcium metabolism disorders among HIV infected individuals.

## Abbreviations

AIDS: Acquired immunodeficiency syndrome; ART: Antiretroviral treatment; BMD: Bone mineral density; CI: Confidence interval; HIV: Human immunodeficiency virus; IP: Intestinal parasitoses; LPS: Lipopolysaccharide; OIs: Opportunistic infections; OR: Odds ratio; RANKL: Receptor of activated NF-kappa B ligand; SD: Standard deviation; SPSS: Statistical package for social sciences; TNF: Tumor necrosis factor; WHO: World health organization.

## Competing interests

The authors declare that they have no competing interests.

## Authors’ contribution

AK and TY were involved in the design of the study and carrying out the data collection while BA and BM were involved in data analysis and drafting the manuscript. All authors read and approved the final manuscript.

## Pre-publication history

The pre-publication history for this paper can be accessed here:

http://www.biomedcentral.com/1471-2458/14/679/prepub
